# Digital interventions in medication adherence: a narrative review of current evidence and challenges

**DOI:** 10.3389/fphar.2025.1632474

**Published:** 2025-10-10

**Authors:** Zoe Moon, Jane Walsh

**Affiliations:** ^1^ Centre for Behavioural Medicine, School of Pharmacy, University College London, London, United Kingdom; ^2^ School of Psychology, University of Galway, Galway, Ireland

**Keywords:** adherence, interventions, digital, eHealth, mHealth

## Abstract

Non-adherence to prescribed treatments remains a major challenge facing the healthcare system. Despite decades of research, interventions to improve adherence typically have not shown large or sustained effects on adherence and are rarely implemented. Digital technologies provide a potential platform to increase the reach and cost-effectiveness of adherence interventions, allowing them to be widely rolled out. Current evidence suggests that digital interventions can increase adherence, but results are mixed with many interventions failing to improve adherence. This is likely because whilst the included interventions all utilise digital platforms, they vary significantly in their design, content and delivery. Many interventions are not theory or evidence based, do not include patient or healthcare practitioner involvement or focus simply on providing reminders. Evidence suggests that well-designed interventions which are evidence-based, are personalised and maximise interactivity are more likely to be successful. These well-designed interventions hold promise for improving adherence at scale. This narrative review discusses the current challenges facing digital adherence interventions and describes barriers to implementation or adoption which need to be resolved. These include considering reach, accessibility, and acceptability, to avoid increasing existing health inequalities. It is also critical to consider the quality, safety and regulation of available apps and other digital tools, as well as investigating ways to enhance engagement and retention. Finally, some digital tools may require integration into existing systems or may necessitate training of relevant staff. Overall, digital interventions appear to be a promising tool for improving medication adherence, but further work is needed to optimise these tools.

## 1 Introduction

The World Health Organization has classified treatment adherence as a major global problem (Burkhart and Sabaté, 2003). It is estimated that 20%–50% of patients do not take their medication as prescribed (Bosworth et al., 2016). The reasons patients do not take their medication correctly can be either unintentional, such as confusion or simple forgetfulness (Mira et al., 2015), or intentional, where the patient makes a deliberate decision not to take their treatment (Horne et al., 2019). Support to improve adherence therefore needs to foster both motivation and ability to adhere (Horne et al., 2019).

Digital technologies are increasingly being used to deliver these interventions, due to the proliferation of smart phones and other technology developments globally. Internet access continues to grow, with an estimated 5.44 billion internet users worldwide in 2024, accounting for two-thirds of the global population (Statista, 2024b). 69% of the global population have access to a smart phone (Statista, 2024c), with very high levels of penetration in the United Kingdom (84% (Statistics, 2020)) and US (90% (Center, 2024)). Engagement with and implementation of digital healthcare has also been rising over the past 10 years, particularly since the COVID-19 pandemic in 2020 (Mahajan et al., 2021; Mosnaim et al., 2020).

These figures highlight the potential for delivering health programs such as adherence interventions through a mobile phone, computer or similar device. These technologies are often very cost-effective and can reach large numbers of patients with little effort, as well as enhancing the potential for personalisation and automation of interventions.

This narrative review will first provide an overview the current evidence base for digital interventions to improve adherence, followed by an outline of issues in the field and factors associated with the success of digital health adherence tools.

## 2 Current evidence base for digital interventions

### 2.1 Text messaging

Several systematic reviews of studies including both randomised and non-randomised designs have shown that Short Message Service (SMS) text interventions can improve medication adherence in patients with diabetes, hypertension and/or dyslipidemia (Belete et al., 2023; Bingham et al., 2021). However, other systematic reviews including just Randomised Controlled Trials (RCTs) with a greater number of studies show more mixed results (Bond et al., 2021; Redfern et al., 2024). For example, a recent Cochrane review of RCTs evaluating text messaging for medication adherence in secondary prevention of cardiovascular disease concluded that the evidence was very uncertain, with only 10 out of 18 studies showing a beneficial effect on adherence compared to usual care (Redfern et al., 2024). Furthermore, any effects tend to be very short term, or do not persist past the end of the intervention (Mulawa et al., 2018; Gautier et al., 2021), and issues have been raised with study quality, such as issues with blinding and selective reporting (Palmer et al., 2021; Adler et al., 2018). Despite these mixed effects, one review found that of the studies (13 of 18) who reported user feedback, satisfaction and interest was very high (Bond et al., 2021).

Some studies have attempted to identify the best ways of optimising text message interventions. Whilst no studies have directly compared tailored vs generic messaging to enhance medication adherence, text message interventions which go beyond simple reminders and are tailored to the individual’s beliefs have shown success in improving adherence (Petrie et al., 2012; Riaz and Jones Nielsen, 2019) and tailored messages have been shown to be more effective in changing other health behaviours (Head et al., 2013). Interventions lasting 6 months or longer were more effective than those that are shorter term (Belete et al., 2023), and tapering for an additional 3 months has also shown to be useful in maintaining adherence after the initial intervention (Belzer et al., 2025).

### 2.2 Apps and web-based programs

Many health behaviour change interventions are now being delivered through mobile applications (apps) or other web-based programs. These allow for the delivery of content direct to the user, along with enhanced options for personalisation, interaction and reminders. A review in 2015 found 681 available adherence apps on the Apple App Store or Google Play Store (Ahmed et al., 2018). The number of adherence apps has grown since then, with a 2017 review estimating 800 medication management apps available (Dayer et al., 2017), and a 2024 review finding 53 available health apps in asthma alone (Robinson et al., 2024). However, despite this proliferation of available apps, there is little consistent evidence for their efficacy (Chong et al., 2023; Cao et al., 2024). Clinical studies evaluating the impact of apps on improving adherence have shown mixed results (Aungst, 2021). A 2020 review of 9 trials of mobile medication adherence apps across a range of health conditions found a significant pooled effect, but five of the nine included studies did not report a significant effect on adherence (Armitage et al., 2020), potentially due to variability in adherence measurement, techniques used and the extent of the tailoring within the included apps.

This lack of consistent evidence for apps is likely due to the significant variation in design, content and delivery (Ng et al., 2020). Few of the publicly available apps meet relevant criteria for quality, content or functionality (Masterson Creber et al., 2016) and most lack a sufficient evidence base. Reviews of trials suggest those that include more interactive features, such as interaction with medical providers, social networking and gamification features, tended to be more effective (Unni et al., 2018; Cazeau, 2021), yet these features are often lacking from publicly available apps (Wang et al., 2024). Similarly, it has been suggested that tailoring intervention content to the individual user will also be associated with positive effects on adherence (Armitage et al., 2020; Goradia et al., 2021; Stewart et al., 2023). For example, several digital interventions which tailor the content to the individual’s medication beliefs have shown success in improving adherence (Lakshminarayana et al., 2017; Chapman et al., 2020; Hughes et al., 2022). In the 2020 review mentioned above, the authors highlight examples of successful highly tailored interventions and state their results may support the hypothesis that level of tailoring is associated with the effectiveness of adherence apps (Armitage et al., 2020).

Recent innovations include the use of gamification in medication adherence apps, including features such as social connectivity, avatars, alternate realities, leaderboards, points and badges. These features are proposed to enhance medication adherence as well as adherence to the app itself (Ahonkhai et al., 2021). A review of five studies using gamification features (e.g., leaderboards, levelling up, quests) to improve medication adherence across a range of conditions, found that three of the five studies showed significant improvements in adherence (Tran et al., 2022). Overall, the evidence base for adherence apps is mixed with many poor quality studies, making it difficult to draw conclusions on their effectiveness (Ng et al., 2020). Reported issues with quality include small sample sizes, self-presentation bias, potential conflicts of interest, lack of appropriate control arms and self-reporting of adherence outcomes (Ng et al., 2020).

### 2.3 Monitoring and smart products

Over the last decade, the popularity of digital medication adherence systems has surged, with both healthcare providers and patients acknowledging their role in enhancing adherence and overall health results. A recent review by Mason et al. (2022) identified a variety of technology applications for monitoring medication adherence, including electronic pill bottles or boxes, ingestible sensors, video-based technology, and motion sensor technology. The common expectation is that these technologies accurately monitor medication adherence and can easily be adopted in patients’ daily lives owing to their unobtrusiveness and convenience of use.

Sensor technologies have been increasingly used to track the medication-taking behaviours of patients. For example, the Medication Event Monitoring System (MEMS) can record every time the patient opens the pill bottle via a sensor embedded in the pill cap. These medication monitors are increasingly used as part of strategies to improve adherence. Despite this, there is limited consensus on how to determine or select the appropriate medication adherence monitoring technology for use. There is a growing need for technology assessment criteria to guide the development and selection of appropriate technologies for monitoring medication adherence to improve patient outcomes (Basu et al., 2019).

Some recent studies have shown promising findings for the use of smart technologies to improve medication adherence. A systematic review by Chan et al. (2022) found that patients receiving an electronic adherence monitoring (EAM) intervention (most commonly devices which record pillbox being opened and sent reminders) had significantly better adherence than those who did not. In this review, data from 27 studies (n = 2,584) were extracted for the adherence outcome, Most studies were conducted on adults (87%) and the most common conditions were in asthma (21%) or human immunodeficiency virus (HIV) (19%), or hypertension (13%). The authors concluded that improved adherence did not consistently translate into clinical benefits (Chan et al., 2022). Acceptability data were mixed, with perceptions of the device being negative in nearly half of the included studies. Issues with acceptability included the reminder beeps, the size of the device and concerns about disclosure. Feedback on the intervention itself was more positive, with patients looking forward to receiving their adherence data. The authors conclude that further research is required to assess patient acceptability and explore effects on clinical outcomes and. A study by van de Hei et al. (2023) found that digital inhaler–based interventions can yield long term cost-savings by optimising medication adherence and inhaler technique and reducing additional biologic prescriptions in patients with difficult-to-treat asthma (van de Hei et al., 2023).

Stakeholders’ expectations regarding the use of health information technology for monitoring medication adherence can also vary. From a clinical practice perspective, a user-friendly interface and the accurate monitoring of adherence are considered when selecting appropriate monitoring technologies. From the technological development perspective, although system accuracy and data fidelity remain high priorities, developers also need to consider the feasibility of technical engineering of the system, such as energy consumption and battery lifetime (Aldeer et al., 2018). Human interactions with these technologies can be complicated owing to the comprehensive medical and pharmacological contexts, as well as multidimensional patient medication adherence behaviours.

### 2.4 Artificial Intelligence and adaptive interventions

Artificial Intelligence (AI)-powered mobile applications are those that apply logical algorithms which are capable of learning from data and making autonomous decisions based on generalizable rules (Zavaleta-Monestel et al., 2025). These have proven to be valuable tools in monitoring and improving medication adherence (Zavaleta-Monestel et al., 2025). In a study conducted by Labovitz et al. (2017), an AI-based smartphone app was developed for stroke patients on direct oral anticoagulant therapy. The app used a neural network to identify the patient and the prescribed drug, confirm ingestion through the phone’s camera, and provide medication reminders. The study found a 100% adherence rate among patients using the app compared to 50% in the control group, and identified positive patient feedback. However, the study was small (n = 28), and therefore further research with larger sample sizes is needed to determine long-term effectiveness. Similarly, Bain and colleagues (Bain et al., 2017) used an AI platform incorporating facial recognition and drug verification for real-time monitoring of schizophrenia patients in a 24-week clinical trial. The study demonstrated 17.9% higher adherence in the AI-monitored group compared to a control group receiving modified direct observation therapy. Another clinical trial used a voice-based conversational AI application to support type 2 diabetes patients (Nayak et al., 2023). The results showed that insulin adherence rates were 32.7% higher in the AI-voice application compared to the standard care group.

AI-driven reminder systems have been developed to encourage medication adherence by sending timely reminders to patients. Brar Prayaga et al. (2018) explored the use of “mPulse Mobile,” an SMS-based AI reminder system in older patients with non-communicable diseases. They observed significantly higher medication refill rates in the group that received AI-generated SMS reminders compared to a control group that did not receive any reminders. A study by Chaix et al. (2019) found that AI can also play an important role in indirectly improving adherence by empowering patients (Chaix et al., 2019). In their study they used “Vik,” a chatbot designed for breast cancer patients to provide personalised health information, including medication reminders. The study showed that patients who engaged more with Vik were observant when using a treatment reminder function, and that medication adherence improved by more than 20% in this group.

AI-assisted technology could also be used to optimise prescriptions by prioritising medications that match the insurance/preferred pharmacy of the patients and check drug–drug interactions. AI has already been shown to be useful for medication reconciliation, which is a procedure often used to reduce medication errors (Long et al., 2016). One of the major contributions that AI-assisted technologies has had in recent years in disease management is through machine learning (ML) and big data analytics. For example, Koesmahargyo and colleagues (2020) used ML to predict medication non-adherence based on real-time dosing data collected from smartphone videos of patients taking their medications (Koesmahargyo et al., 2020). This approach provided highly accurate predictions of adherence across both the trial period and subsequent days or weeks. A systematic review of literature on AI highlighted that machine learning is currently the most commonly used AI technology in healthcare (Guo et al., 2020). In general, however, this field is still in its infancy; there are currently 100 FDA-approved AI/machine learning-based medical devices and algorithms, which are constantly updated on an online database (Medicalfuturist, 2025). A recent review examined the use of AI tools for patient support to enhance medication adherence, with results showing that although the evidence supporting AI tools to assist patients is weak, smart systems using AI tools are promising in helping patients use prescribed medications (Reis et al., 2025). Based on current evidence, AI-powered, personalised approaches are best suited to complex behavioral barriers to intentional adherence, whereas basic digital tools can serve as reminders and educational aids to improve unintentional adherence by providing real-time feedback and tracking.

## 3 Factors associated with success of digital health adherence tools

**FIGURE 1:**
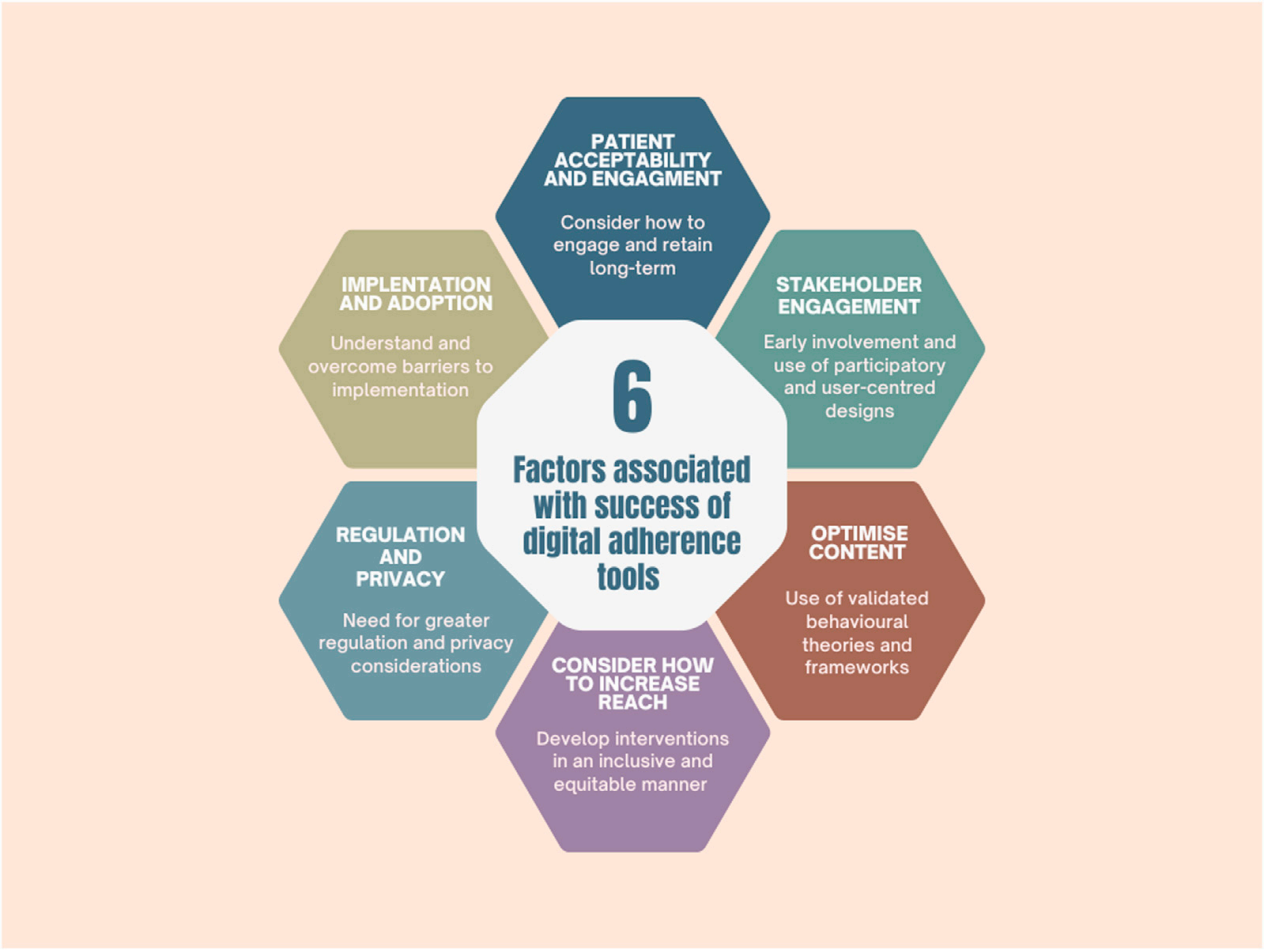
Factors associated with the success of digital adherence tools.

As described above, evidence on the effectiveness of digital adherence interventions is mixed, with many interventions failing to improve adherence. In order to develop interventions which will successfully engage participants and improve adherence, the following factors need to be considered (see Figure 1 for a summary).

### 3.1 Patient acceptability and engagement

Engaging patients in an intervention and retaining them throughout is one of the biggest challenges facing any e-health intervention (Eysenbach, 2005), including adherence interventions, with many participants declining, dropping out of or not fully engaging with adherence interventions (Habib et al., 2021; Ping et al., 2022; Côté et al., 2020). For example, a web-based intervention to support medication adherence in people with HIV found that only 69% accessed the intervention, and of these only 36% completed more than one session. Only four of the initial 45 participants reached session four (Côté et al., 2020).

Reasons for this lack of engagement are complex and multi-faceted. Barriers to e-health in general include concerns about privacy and confidentiality, limited access to the relevant device or the internet, and lack of perceived need for digital support (Moecke et al., 2024; Morrissey et al., 2018). With regards to adherence interventions, concerns around privacy and data ownership are particularly relevant in interventions involving monitoring or any form of AI, with participants reporting concerns that this data could be used against them (e.g., with insurance companies) (Klugman et al., 2018). Technical issues or lack of user-friendly designs may also impede engagement to digital interventions (Ping et al., 2022; Grindrod et al., 2014). Older adults may face physical difficulties such as issues using small buttons on smartphones, reading small fonts or hearing notifications (van Acker et al., 2023). To overcome these issues, it is essential to involve the target population in intervention development and identify barriers relating to engagement and trust. For example, a survey of medication reminder app users in Singapore suggested that highlighting how apps protect personal data or offering anonymous usage should increase app usage (Ping et al., 2022). Another study found that people were more likely to agree to use an app if a clinical staff member would help them (Morano et al., 2019).

Usability testing and stakeholder feedback can help to develop interventions which are easy to understand and use for the target population (Grindrod et al., 2014; Hosszú et al., 2024). For example, Blixen et al. (2018) collected user feedback as part of the development of a text messaging intervention to improve adherence in people with bipolar disorder and hypertension (Blixen et al., 2018). Results highlighted key areas to increase patient acceptability, such as customising messages, writing out in full instead of abbreviated text speak and a focus on positive rather than negative messages. Similarly, user testing of a web-based diabetes adherence intervention identified several errors and provided recommendations on how to improve the site’s user interface (Nelson et al., 2016). Applying user experience (UX) principles, such as clear instructions and user-friendly error messages is also essential in developing apps which are intuitive and that people do not get frustrated with and stop using (Omaghomi et al., 2024). Gamification features such as rewards systems, points and leaderboards may also increase engagement (Omaghomi et al., 2024), as does the overall aesthetic and appearance of the app (Michie et al., 2017). Users also report engaging more with apps that appear to be credible, that are personalised and that allow communication with other users or HCPs (Michie et al., 2017). For example, a personalised smartphone based tracker app in Parkinsons disease showed significant improvements in adherence (Lakshminarayana et al., 2017). Analysis of usage found that 72% of participants in the intervention group continued to use and engage with the application across the 16 week period, with most using the app almost every day. However engagement with apps does not always lead to improved adherence. For example, a gaming app to improve medication in rheumatoid artritis found no significant improvements in adherence, despite the fact that 79% installed the game and 65% of these were active for at least 30 days out of 90 (Pouls et al., 2022).

### 3.2 Stakeholder engagement

Engaging end-users and wider stakeholders in the early design of interventions is essential to ensure participant acceptability, engagement and retention. Participatory approaches to digital health research have received increasing attention over the past 2 decades, particularly with regards to their role in developing effective digital interventions to promote medication adherence. Public and patient involvement (PPI) refers to the process of involving members of the public or patient groups in the research or design process. This involvement can occur at different stages of the research or design process. One of the driving motivations behind participatory approaches such as PPI is the idea that, in the case of public health research, members of the public have a right to input into designs and decisions in the context of research which may affect them (Bagley et al., 2016). Involving stakeholders who will interact directly or indirectly with the outcomes of research, tool design, or interventions, serves to ensure that the research is relevant, conducted in an ethical and acceptable manner, and that the research is designed in a participant-friendly or user-friendly manner.

In the past, mHealth tools have commonly been designed with consideration only given to existing healthcare systems and protocols, with little or no involvement of the end-users (Schnall et al., 2016). Increasingly however it is recognised that, in order for mHealth tools and applications to be effective, careful consideration needs to be given to the needs, requirements, and capacities of the end-users. Some reported barriers and enablers such as the importance of data privacy and security appear to be unique to PPI in digital innovation and these need to be addressed as part of this process (Baines et al., 2022).

A strong emphasis on participatory research and user-centred design, are said to play a key role in overcoming the uptake and retention issues described previously (Morton et al., 2020). While PPI focuses primarily on the involvement of patients in research and design, participatory approaches may involve engagement with stakeholders across various levels of healthcare delivery, depending on the purpose of the research design. Involving stakeholders, such as community health workers, nurses, administrators, and data managers in the design of mHealth tools allows for the gathering of valuable input in relation to various factors in effective design, including relevance, usability, and acceptability (de Beurs et al., 2017; Brewer et al., 2020). Effective eHealth interventions for self-management involve multidisciplinary teams harnessing diverse expertise. Systematic frameworks for intervention design and evidence-based user-centred methods, such as the person-based approach and Public and Patient Involvement (PPI) (Baines et al., 2022) facilitate this teamwork.

The WHO underscores involving end users in initial design phases to inform critical elements like perceived benefits and barriers to behaviour change, aligning interventions with community characteristics (WHO, 2021). Recognising this, increased emphasis has been placed on early involvement of users and stakeholders. The person-based approach leverages in-depth qualitative research to define guiding principles and key intervention features, essential across development stages, including planning, testing, and clinical evaluation. It aligns with in-depth approaches from information systems and human-computer interaction, emphasising understanding user knowledge, behaviour, motivations, and cultural contexts. Traditional user-testing focuses on utility and engagement, aiming to enhance technology usage. In contrast, the person-based approach, rooted in health psychology, targets behaviour change techniques and their implementation to boost participant engagement, driving intended outcomes.

### 3.3 Optimised content–use of behavioural theories/frameworks

Behaviour change theories can be used to aid the development of interventions to address relevant barriers to adherence and identify solutions for improving adherence. There are many long-standing, influential theories, including the Theory of Planned Behaviour (Ajzen, 1991), Goal-Setting Theory (Locke and Latham, 2015), the Health Belief Model (Janz and Becker, 1984), and Bandura’s (1986) Self-Efficacy Theory (Bandura, 1982). The Perceptions and Practicalities Approach (PaPA (Horne et al., 2019)) is a behaviour change theory developed specifically to understand non-adherence. The United Kingdom National Institute for Health and Care Excellence (NICE) guidelines for Supporting Adherence (Nunes, 2009) recommend the application of the PaPA, suggesting that any adherence support needs to consider the perceptual factors (e.g., beliefs about illness and treatment) that influence motivation to take a prescribed treatment, as well as the practical factors influencing ability to take the treatment (Horne et al., 2019). Evidence suggests that interventions which address both perceptual and practical factors influencing adherence are more likely to succeed. For example, a review of interventions to improve adherence to antiretroviral therapy found that interventions which addressed individuals’ specific perceptual and practical barriers to adherence were more effective than those that just addressed practical barriers like forgetting (Zoe Moon et al., 2023).

Another approach is the Behaviour Change Wheel (Michie et al., 2011) developed by synthesising 19 different frameworks of behaviour change. The Behaviour Change Wheel provides a useful way of linking a model of behaviour to common functions of interventions to change that behaviour (e.g., education, persuasion, coercion, incentivisation), and in turn, linking these intervention functions to policy categories (e.g., service provision, guidelines) that facilitate behaviour change. In addition, the Behaviour Change Technique Ontology (BCTO) (Marques et al., 2023) promotes the use of Behaviour Change Techniques (BCTs), defined as the observable, replicable components of behaviour change interventions. The BCTO provides a standard terminology and comprehensive classification system for the content of behaviour change interventions that can be reliably used to describe interventions. The techniques included in the ontology have been synthesised from related constructs drawn from theories and frameworks across clinical and health psychology research and practice. Using the BCT ontology to design effective interventions is therefore not inconsistent with other theoretical approaches.

Kahwati and colleagues used the BCT Taxonomy to conduct a qualitative comparative analysis of a systematic review of 60 complex interventions to identify combinations of BCTs that were most effective for improving medication adherence in outpatients with chronic conditions. Improvement in adherence was reported in more than half of the studies (57%). Of these studies, there were seven different configurations of BCTs that increased adherence. However, the most common and efficacious combination of techniques was ‘increasing knowledge’ coupled with ‘increasing self-efficacy’ (Kahwati et al., 2016). A content analysis of the BCTs present in 166 available apps reported that 12 of a possible 96 BCTs were present across these apps, and that 96% of the apps included the BCTs of ‘action-planning’, and ‘prompting/cues’. More than one-third of the apps that were reviewed featured the BCTs ‘self-monitoring’ and ‘feedback on behaviour’ (Morrissey et al., 2016).

### 3.4 Reach and inequalities in access

It has been suggested that digital technologies hold great potential for offsetting health inequalities, by increasing access and reaching those who may not traditionally receive support (Sharma and Patten, 2022; van de Vijver et al., 2023). However, there is also the potential for digital technologies to widen existing health inequalities, causing a “digital divide”, should they not have equitable reach or effectiveness (Latulippe et al., 2017). For example, digital health literacy and internet access are reported to be lower in underprivileged populations such as immigrants and individuals with lower socioeconomic status or less formal education (Estrela et al., 2023). This is of concern as these are groups who are already facing health inequalities.

Research has highlighted differences in terms of who has access to relevant digital devices. A survey of 2009 women with breast cancer in the United Kingdom found that 20% did not have access to a Tablet or Smartphone, and that the women without access were more likely to be older, have less formal education and be from a more deprived area (Moon et al., 2022). In the US, whilst 97% of college graduates own a smartphone, this falls to 83% in people with no college education (Center, 2024). In the United Kingdom, 96% of the highest socioeconomic group are smartphone users compared to 84% of the lowest socioeconomic status group (Statista, 2024a). Across the world, a UN report cited in the least developed countries only 27% of the populations are Internet users (Nations, 2023).

However, access to the internet, smartphones or other devices is only one part of the picture. It is also important to consider whether there are any factors influencing willingness to engage with digital health interventions. For example, studies report that older adults, those who are less highly educated and people from minority ethnic groups are less likely to be users of mobile health apps or to seek health information online (Bol et al., 2018; Fareed et al., 2021). Receptivity towards mobile phone text messages as a healthcare intervention also reduces with increasing age, and lower education and income levels (Serrano et al., 2016). Specifically with regards to digital adherence interventions, a US study showed that people with diabetes with lower health literacy and who were not of white ethnicity were less likely to participate in the intervention (Nelson et al., 2016). However, engagement did not differ by age, gender, education, income or health literacy, suggesting fairly wide reach. In another study, people with HIV who had less formal education were less willing to adopt mobile phone technology to improve their adherence (Morano et al., 2019).

Taken together, these studies support the idea that digital health interventions may be less likely to be accessed or used by people from lower socioeconomic status backgrounds, which is of concern given the existing health inequalities in these groups. However, some other studies have shown that patients in diverse or low-income communities show greater interest in mHealth apps than those from white or high-income communities (Humble et al., 2016; Ramirez et al., 2016).

Research has also highlighted potential differences in how effective digital behaviour change interventions are for different groups of people. Whilst this has not been explored in adherence specifically, a systematic review and meta-analysis of digital behaviour change interventions for physical activity found that the interventions were effective in those with high socioeconomic status but not in people of low socioeconomic status (Western et al., 2021). Therefore, attention may need to be paid to understanding whether the benefits of adherence interventions are equitable across all participants.

More research is warranted to fully understand whether adherence digital interventions will further the “digital divide” or help to close existing gaps. However, regardless, intervention developers need to be mindful of developing interventions in an inclusive and equitable manner. Several guidelines have been developed to assist with this (Latulippe et al., 2017; Miller et al., 2023), as well as the Carnegie United Kingdom Trust 12 recommendations for eliminating digital exclusion (Georgina Bowyer, 2020). Key elements of these guidelines include ensuring universal access to the tool, co-creating with a diverse and relevant stakeholder groups, accounting for varying levels of health literacy, and collecting quality data to monitor access and engagement.

### 3.5 Regulation and privacy

Rapid developments in digital technology has far outpaced regulatory bodies’ capacity to address issues around quality, data regulation and privacy. A study by Backes et al. (2020) investigated whether healthcare providers could safely recommend mobile health apps to their patients to promote medication adherence (Backes et al., 2020). In their study they evaluated eligible apps and concluded that none of the apps had undergone a process for certification, little information was provided on security and data protection and that more clinical studies with chronic patients are necessary to measure long-term app impacts. The authors suggest that some of these shortcomings might be corrected through the introduction of General Data Protection Regulation (GDPR) in the European Economic Area (EEA) and more scrutiny through regulatory bodies in the EU/EEA and the United States. They further concluded that none of the applications should be recommended by healthcare providers.

Research by Grundy et al. (2019) found that the accuracy and quality of information provided within medical and health apps cannot easily be ascertained and these factors are likely to affect medication adherence and, more importantly, patient health outcomes (Grundy et al., 2019). They found that apps often involve communicating patient-specific data over the internet which raises the issue of patient privacy. They further reported that up to 80% of mobile health apps transmit user-related information to online services and 66% of apps sent unencrypted identifying information over the Internet. They conclude that the benefits of secure communication of information between health providers and patients cannot be ignored.

Magrabi et al. (2019) examined the challenges around regulation of apps to promote medication adherence and concluded that an evidence-based approach that is informed by the current landscape of health apps is required (Farah et al., 2019). They suggest that operational oversight and surveillance could be considered at a national and regional level using common frameworks so that it is possible to compare patterns over time and between settings, and to develop and prioritise preventive and corrective strategies. A professional foundation for regulation of such technologies would permit more widespread use of evidence-based apps to promote medication adherence. Finally, the role of citizen developers should also be considered within this digital health ecosystem.

### 3.6 Implementation and adoption

A final issue with digital adherence interventions is that they are often under-utilised and few are implemented at scale (Kardas et al., 2022). Trials of adherence interventions tend to fail to consider factors relevant to implementation into real-world settings (Kostalova et al., 2022), and reviews have concluded that the long-term sustainability and feasibility of digital adherence interventions remains to be determined (Chan et al., 2022; Griffee et al., 2022). Barriers to the successful implementation of eHealth interventions in general include cost, increased workloads, lack of healthcare professional motivation, issues with interoperability, and lack of suitable infrastructure, training and support (Kardas et al., 2022; Ahmed et al., 2019; Granja et al., 2018; Chimweta et al., 2025). Particularly in the developing world, issues with local telecommunication networks may act as a barrier to intervention implementation (O'Connor et al., 2022). Across all contexts, acquiring the funding for ongoing maintenance and hosting can be a barrier to implementation and utilisation (Ahmed et al., 2019). Implementation science can provide useful insights and should be considered from the start of any project to ensure that the digital adherence interventions developed have a chance of being implemented (Kostalova et al., 2022; Zullig et al., 2019). Issues around reimbursement are also a barrier to implementation, and more data on long-term clinical effects and cost-effectiveness may be needed to overcome this (Kardas et al., 2022; Borah et al., 2025).

## 4 Conclusion

Digital technologies have emerged as a promising tool for addressing the significant global issue of medication non-adherence. However, the evidence supporting the effectiveness of these digital interventions is mixed, with many studies showing inconsistent or short-term improvements in adherence. This variability is largely due to the diverse designs, content, and delivery methods used in digital tools, many of which lack a strong evidence base or user-centered design. While digital interventions have the potential to reduce healthcare costs and improve medication adherence, careful attention must be paid to ensure these technologies do not inadvertently widen existing health inequalities. Addressing the “digital divide” by ensuring equitable access, usability, and acceptability across diverse populations is essential to prevent exacerbating disparities in healthcare access and outcomes.

Future interventions aimed at improving medication adherence should emphasise personalised approaches that consider individual patient needs, beliefs, and preferences. Leveraging AI and machine learning algorithms can enhance engagement and effectiveness by tailoring content, reminders, and feedback. Incorporating interactive elements, such as communication with healthcare providers and peer support networks, can further boost adherence. Rigorous evaluation and the establishment of quality standards are essential, with a focus on long-term outcomes, patient engagement, and clinical benefits through well-designed clinical trials.
